# Immunohistological study of the effect of vascular Endothelial Growth Factor on the angiogenesis of mature root canals in rat molars

**DOI:** 10.1590/1678-7757-2017-0437

**Published:** 2018-05-11

**Authors:** Cleber Keiti Nabeshima, José Edgar Valdivia, Hector Caballero-Flores, Victor Elias Arana-Chavez, Manoel Eduardo de Lima Machado

**Affiliations:** 1 Universidade de São Paulo, Faculdade de Odontologia, Departamento de Dentística, São Paulo, São Paulo, Brasil.; 2 Universidade de São Paulo, Faculdade de Odontologia, Departamento de Biomateriais e Biologia Oral, São Paulo, São Paulo, Brasil.

**Keywords:** Angiogenesis, Vascular endothelial growth factor, Endodontics

## Abstract

Tissue bioengineering has been applied to Endodontics to seek a more biological treatment. The presence of blood vessels is crucial for cell nutrition during tissue formation.

**Objective:**

This study analysed the application of vascular endothelial growth factor (VEGF) in the angiogenesis of mature root canals.

**Material and methods:**

Upper first molars of twelve 13-week old Wistar male rats were used. The root pulp of the mesiobuccal canal was removed and the root canal instrumented with K-files up to size #25. Periapical bleeding was induced into the root canal by introducing a #15 K-file beyond the apex. The teeth on the right side of the arch were filled up with blood clot (G1), whereas those on the left side were filled up with blood clot plus 50 ng/ml of VEGF (G2). Teeth were sealed with light-curing glass-ionomer cement and the animals were sacrificed after 60 days. The maxilla was dissected and fixed before obtaining serial sections for histological processing with haematoxylin-eosin (HE) and immunohistochemical factor-VIII. Immunohistochemical labelling was evaluated using scores for statistical analysis.

**Results:**

Immunohistological analysis demonstrated the presence of angiogenesis in both groups, but with higher angiogenic maturation in G2 during the experimental period (p<0.05). HE staining showed connective tissue with absence of odontoblasts in all specimens.

**Conclusions:**

It can be concluded that it is possible to obtain angiogenesis in mature root canals with or without the use of VEGF, although the latter tends to accelerate blood vessel formation.

## Introduction

Regenerative dentistry is one of the investigative fields that promises to change current paradigms by relying on bioengineering principles. Since the discovery of stem cells in the dental pulp, Endodontics has been highlighted in this area, and the use of an intracanal blood clot for pulp revascularisation has been proposed^3^ ^,^ ^16^. However, most experiments have focused on the treatment of the permanent immature teeth, while the therapeutic approach on mature root canals has been little explored.

Although bioengineering comprises the triad of cells, scaffold and growth factors^23^, vascularisation is crucial for tissue development^9^ ^,^ ^26^. Thus, angiogenesis is a key element in the new tissue formation so that cells may receive the nutrients necessary to their proliferation and differentiation.

Among the several growth factors, the vascular endothelial growth factor (VEGF) has been indicated as one of the main ones involved in angiogenesis^14^. In addition, VEGF promotes cell proliferation, differentiation and has chemotaxis towards endothelial cells and homing cells^13^ ^,^ ^18^ ^,^ ^28^. However, studies confirming this information were performed on culture cells or subcutaneous implantation. Ruangsawasdi, et al.^25^ (2016) demonstrated that the site can affect pulp regeneration. Considering that there are no *in loco* studies evaluating the angiogenesis on mature root canals, this study aimed to analyse the effect of VEGF on the angiogenesis of mature root canals in rat molars.

## Material and methods

After approval by the Animal Ethics Committee (CEUA #018/2014), twelve 13-week old Wistar male rats (*Rattus norvegicus albinus*) were intramuscularly anesthetised with 2% xylazine (Anasedan, Ceva; Paulínia, SP, Brazil) and 10% ketamine (Dopalen, Ceva; Paulínia, SP, Brazil) at a ratio of 1:1 and dose of 0.1 ml/100 g. The pulp chambers of upper first molars were accessed by using a ½-inch carbide round burr at low rotation (Microdont; São Paulo, SP, Brazil). The mesiobuccal root canal was located with a #10 K-file (Dentsply Maillefer; Ballaigues, Vaud, Switzerland) and then instrumented it up to a #25 K-file in association with 1.8 ml of 2.5% sodium hypochlorite (Fórmula e Ação; São Paulo, SP, Brasil). Final irrigation was performed with 1.0 ml of 2.5% sodium hypochlorite followed by 1.0 ml of 17% EDTA (Fórmula e Ação; São Paulo, SP, Brasil).

The apex was opened using a #8 K-file and then widened it to a #20 size. Periapical bleeding was induced by inserting a #15 K-file beyond the apex.

Lyophilised VEGF-165 (Recombinant Rat Vascular Endothelial Growth Factor; Prospec; Ness-Ziona, Central District, Israe^l^) was reconstituted (100 μg of lyophilised VEGF to 1.0 mL of Milli-Q water) and a concentration of 50 ng/mL (1.0 μL of VEGF reconstituted in 1999 μL of Milli-Q water) was used, according to Gonçalves, et al.^9^ (2007).

The teeth were divided into two groups (n=12) as follows:

Group 1 (control): periapical bleeding was stimulated into the root canal and then left it at rest for 5 minutes to form a clot;

Group 2: clot formation was stimulated as described in Group 1, then 5 μL of VEGF was prepared at a concentration of 50 ng/mL and put it into the pulp chamber using a micropipette (0.5-10 μL, Termo Fisher; Waltham, Massachusetts, USA), taking it to the apex with a #10 K-file to be incorporated into the clot, which was left at rest for further 5 min.

Other non-instrumented root canals in the same tooth served to verify the histological features of the vital pulp without treatment.

The coronal access was sealed with light-curing glass-ionomer cement (Vitemer, 3M; Saint Paul, Minnesota, USA). After 60 days, the animals were euthanized and the specimens processed for histological analysis.

The maxillae were dissected and fixed in 0.1% glutaraldehyde plus 4% formaldehyde in 0.1 M phosphate buffer at pH 7.4, placed in a microwave oven (Pelco 3400, Ted Pella; Redding, California, USA) with air-bubble agitation (cooler pump Pelco 3420, Ted Pella; Redding, California, USA) under controlled temperature of 37°C, and then left at 4°C overnight^17^.

Afterwards, the specimens were decalcified in 4.13% EDTA at pH 7.2 under agitation for 40 d, dehydrated in ascending concentration of ethanol, diafanised in xylol and embedded in paraplast (Sigma-Aldrich; Saint Louis, Missouri, USA). Five-μm-thick transversal serial sections of the coronal, middle and apical thirds were obtained and stained with HE or immunohistochemically labelled. Five sections were placed on a microscope slide for HE staining and five sections were placed on a silanized slide for immunohistochemistry, and so on until the end of the tooth. Approximately 20 slides with 5 sections were obtained *per* third – 10 slides for HE and 10 slides for immunohistochemical labeling. Then, the middle slide was used for the evaluation. The slides were paired for HE and immunohistochemical processing.

The immunohistological processing was performed by using a DAKO Cytomation EnVision + System HRP rabbit (AEC) kit (K4008, Dako; Carpinteria, California, USA). The sections were deparaffinised by heat at 60°C and immersion in xylol. After using descending concentrations of ethanol, the antigen retrieval was carried out using citrate buffer at pH 6 and 95°C. The slides were incubated with rabbit anti-factor VIII polyclonal antibody (1:300; Bioss; Woburn, Massachusetts, USA) for 6 h at room temperature, followed by secondary antibody (Labelled polymer - HRP, anti-rabbit, Dako; Carpinteria, California, USA) for 30 min. Finally, AEC chromogen solution was applied for 30 min (AEC + substrate chromogen, Dako; Carpinteria, California, USA). Mayer's haematoxylin (Sigma-Aldrich; Saint Louis, Missouri, USA) was used for counter-staining.

As for the immunohistochemical control group, second upper molars were used and processed as described.

All sections were viewed under light microscope (BX60, Olympus Optical; Tokyo, Kantō, *Japan*) at magnifications of 40×, 100×, 200× and 400×. Morphological analysis was performed with HE staining. In addition, the sections submitted to immunohistochemical processing were analysed based on the negative or positive labeling of the angiogenic antibody, so that the intensity labelling (weak and severe) could be observed in the different thirds. Mature functional vessels in the sections presenting negativity were confirmed by analysis with HE staining. The score was assigned based on labelling^19^: 1 (severe), 2 (weak), and 3 (no staining). Kruskal-Wallis test was used for intragroup analysis (thirds), and the Wilcoxon test for intergroup analysis. The level of significance for all analyses was set at p<0.05.

## Results

**Figure 1:**
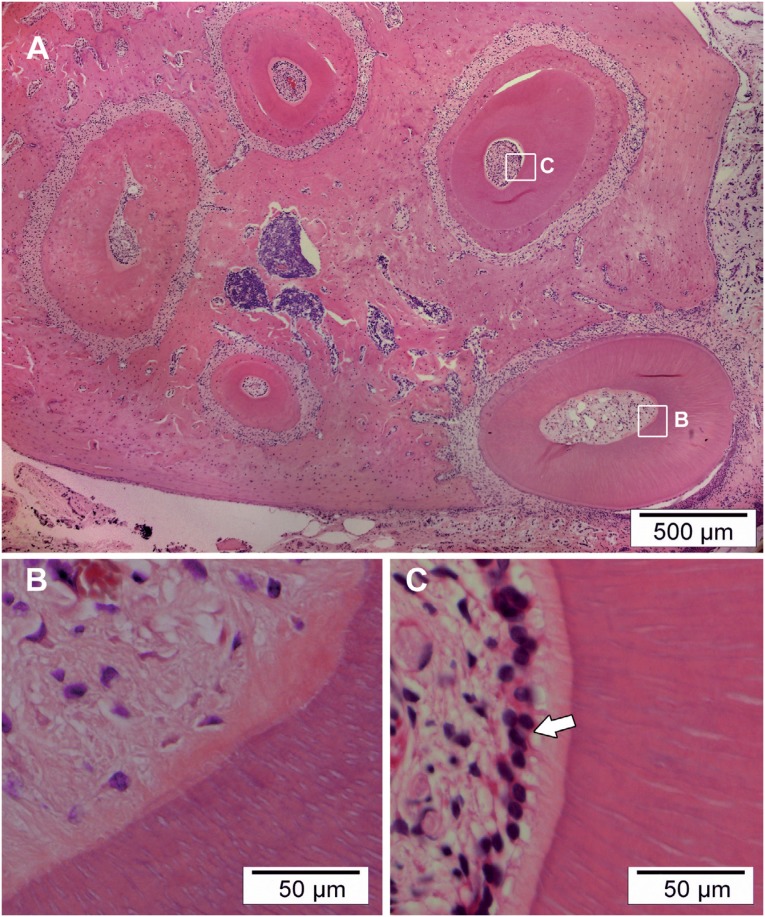
In (A) photomicrograph showing all root canals of the upper first molar in transversal cross-section. In (B) treated mesial buccal root canal showing formation of loosen connective tissue without presence of odontoblasts. In (C) non-instrumented root canal showing vital pulp characterized by the presence of the layer of odontoblasts. Hematoxylin & eosin (HE)-stained

The morphological analysis has shown formation of loosen connective tissue without presence of odontoblasts in all specimens (Figure 1B), differently from pulp in non-instrumented root canals (Figure 1C).

**Figure 2:**
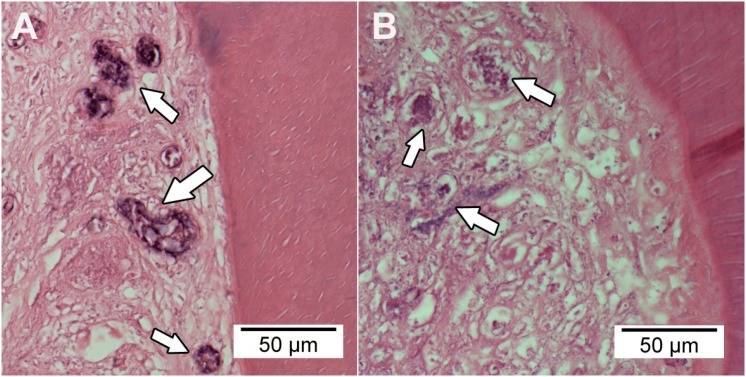
Photomicrograph of the tissue formation in the root canals treated with blood clot without (A) or with vascular endothelial growth factor (VEGF) (B). The arrows indicate necrotic tissue. Hematoxylin & eosin (HE)-stained

In both groups, most specimens showed necrotic tissue surrounded by collagen fibres in all thirds, although necrotic spots were more evident in group 1 than in group 2 (Figure 2).

Some specimens showed formation of highly cellular and well-developed tissue with presence of fibroblasts and functional vessels filled with blood cells. Such a situation was seen in the three thirds of the specimens from the group treated with VEGF, even though this finding was not observed in any section in the cervical third in the group treated with blood clot only.

Immunohistological analysis has demonstrated positivity in the newly formed tissue in most of specimens from both groups and thirds.

**Figure 3:**
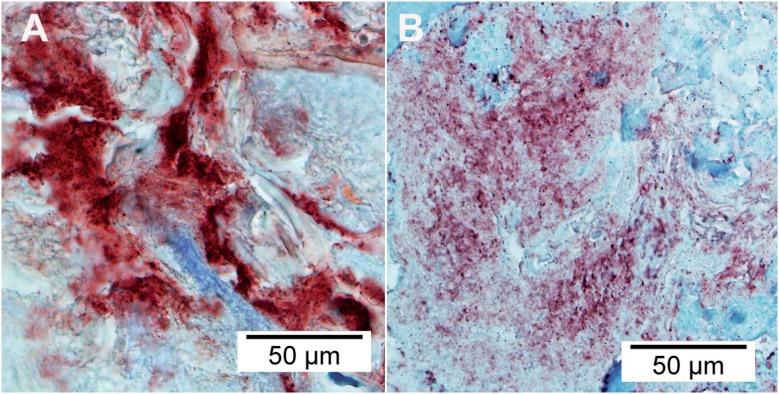
Photomicrograph of the angiogenisis in the root canals. Severe labelling representing the group treated with blood clot only (A) and weak labelling representing the group treated with vascular endothelial growth factor (VEGF) (B). Immunohistological labelled

A prevalence of severe labelling in the coronal and middle thirds was observed in the group treated with blood clot only (Figure 3A). Negativity was present in the middle and apical thirds of only one specimen in this group, which was not observed in the coronal third.

There was predominance of weak labelling in all thirds in the group treated with VEGF (Figure 3B), although severe and negative labelling were also observed. Negativity was present in more than one specimen in this group, although not prevalent. The labelling results according to the group and thirds are shown in Table 1.

**Table 1:** Labelling found in the samples according to the group and thirds

Labelling	blood clot			blood clot + VEGF		
	Coronal (%)	Middle (%)	Apical (%)	Coronal (%)	Middle (%)	Apical (%)
Severe	10 (83.3)	9 (75.0)	5 (41.7)	3 (25.0)	2 (16.7)	1(8.3)
Weak	2 (16.7)	2 (16.7)	6 (50.0)	7 (58.3)	8 (66.6)	6 (50.0)
No labelling	0 (0)	1 (8.3)	1 (8.3)	2 (16.7)	2 (16.7)	5 (41.7)
Total	12 (100)	12 (100)	12 (100)	12 (100)	12 (100)	12 (100)

The statistical test did not demonstrate significant difference between the thirds in both groups (p>0.05), but the group treated with VEGF showed higher angiogenic maturation than the group treated with blood clot only (p<0.05).

**Figure 4:**
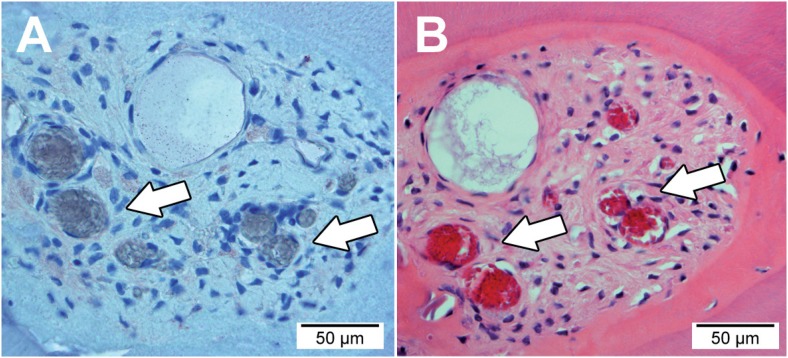
Photomicrograph showing highly cellular and vascularised tissue in the root canal. In (A) transversal cross-section showing negative immunohistological labelling. In (B) transversal cross-section stained by hematoxylin & eosin. Arrows indicate functional vessels

Highly cellular and vascularised tissue in the specimens presenting negativity was confirmed by HE staining (Figure 4).

The immunohistological control showed negativity and a well-organised pulp tissue with presence of functional mature vessels, fibroblasts spread in the pulp and layer of odontoblasts at the peripheral zone.

## Discussion

The immunohistochemical results showed that angiogenesis takes place in mature root canal when periapical bleeding was provoked by instrumentation beyond the apex. The findings also showed that angiogenesis was faster when the VEGF was used to establish a blood clot in the root canal.

Biological Endodontics has been long sought by endodontists, and recent biotechnological advances applied to bioengineering seem to indicate some solutions or alternatives through experiments aiming at pulp regeneration or osteoinduction^13^ ^,^ ^15^ ^,^ ^20^ ^,^ ^23^ ^,^ ^25^. For such, cell culture assays are observed and considered suitable for initial evaluation of substances only as these experiments should not be directly applied to clinical practice because of the lack of previous knowledge^1^ ^,^ ^2^ ^,^ ^4^ ^,^ ^7^ ^,^ ^29^ ^,^ ^30^. However, this approach has limitations, given the absence of immunological complexity of a living organism. *In vivo* experiments have been highly considered and methodologies are based on subcutaneous implants in rats^9^ ^,^ ^13^ ^,^ ^15^ ^,^ ^21^ ^,^ ^23^ ^,^ ^28^, but the connective subcutaneous tissue is highly vascularised and different from that of the dental alveolus. Moreover, assays have demonstrated that it is possible to perform bioengineering studies of tissues in dental tissue *in loco*, thus simulating a clinical situation as much as possible^3^ ^,^ ^6^ ^,^ ^11^ ^,^ ^19^ ^,^ ^20^ ^,^ ^22^. Rats have been used by several authors because they are easy to obtain at low cost, besides reproducing themselves in short periods of time^4^ ^,^ ^9^ ^,^ ^15^ ^,^ ^20^ ^,^ ^21^ ^,^ ^23^ ^,^ ^26^. In addition, the rat metabolism is accelerated, resulting in shorter experimental times and faster responses than studies conducted with humans.

Among the several types of VEGF, the VEGF-A was chosen because of its higher specificity for angiogenesis-related receptors^10^, and the VEGF-165 subtype because of its higher mitogenic potential on endothelial cells^10^ ^,^ ^14^. In addition, it followed previous assays reporting positive results regarding the formation of blood vessels by using this type and subtype of VEGF^4^ ^,^ ^9^ ^,^ ^21^ ^,^.

Several ways have been proposed regarding VEGF use. Aksel, et al.^1^ (2017) used VEGF encapsulated in fibrin gel; Yadlapati, et al.^28^ (2017) described a VEGF-load polymer fiber; and Zhang, et al.^30^ (2014) purposed VEGF gene transfected stem cells. All these studies aimed at a controlled delivery of growth factor to a longer release. However, Aksel and Huang^2^ (2017) observed that continuous VEGF and BMP-2 inhibits the cell differentiation, and on the contrary, that VEGF in the early phase rather than a continuous presence enhances cell differentiation. Thus, this study used direct application of VEGF to blood clot, which resulted in angiogenesis and connective tissue formation such as Yadlapati, et al.^28^ (2017), who applied VEGF delivery system into empty root canal.

Analysis of the results with HE staining has demonstrated the formation of intra-radicular tissue in all specimens, which is in accordance with the literature^3^ ^,^ ^11^ ^,^ ^16^ ^,^ ^28^. Nevertheless, formation of loose connective tissue was observed, differently from other studies reporting dense connective tissue originated from tissue invaginated from the periapical area^3^ ^,^ ^11^ ^,^ ^16^. Therefore, variables such as species used (i.e. animals and humans), experimental time and stage of apical maturation may have affected results.

The presence of necrotic spots in the connective tissue may be related to remnants of non-reabsorbed blood clots, as reported by other authors^11^ ^,^ ^22^. However, another logical explanation might be the presence of remnants of debris from the apical widening. Thus, other authors reported dentine chips or spicules within the newly formed tissue following revascularisation treatment^3^ ^,^ ^16^. In this study, evidence for this scenario was observed in the coronal third, which is a region in which repair is more complex and longer because of its apical distance, given the source of revascularisation^6^ ^,^ ^26^.

The immunohistological analysis showed positivity in the majority of the treated root canals, representing an active process of angiogenesis in both groups. This finding is in accordance with studies reporting positive angiogenesis with or without the use of VEGF applied to bioengineering^4^ ^,^ ^5^ ^,^ ^7^ ^,^ ^9^ ^,^ ^13^ ^,^ ^21^. Vascular formation without application of VEGF may be explained by proteins of extracellular matrix in the dentine, which induce cell proliferation and/or differentiation^15^ ^,^ ^24^ ^,^ ^29^. Studies have indicated that etching dentine with 17% EDTA, as it was performed herein, promotes the release of such proteins and produces better results regarding pulp regeneration^8^ ^,^ ^12^. In addition, growth factors are also present in blood cell concentrates, thus yielding tissue formation^16^.

Both severe and weak labelling patterns were identified and correlated to the stage of angiogenic maturation. Angiogenesis occurs by the action of the growth factor on activating signalling molecules^4^ ^,^ ^27^, which can lead to severe labelling in the initial stimulus of vascular formation. Hence, Moradi, et al.^19^ (2016) also observed strongly positive expressions of factor VIII during angiogenesis, with severe intensity on the first experimental time, showing downregulation at three months postoperative. Thus, the severe labelling is related to the initial stages of angiogenesis, i.e. when most signalling molecules are more active. On the other hand, weak labelling seems to be related to the final stage of angiogenesis, when blood vessels are almost mature. This scenario can be understood in the specimens with negativity, in which mature and functional vessels were present in the HE-stained sections. In fact, these specimens had action of both VEGF and signalling molecules, resulting in angiogenesis and further mature vessel. The immunohistochemical control group confirms that formation of new tissues is due to the treatment applied, since these specimens have no odontoblasts as compared to non-instrumented root canals. Similarly, as for the angiogenic maturation stage, we observed that severe labelling was prevalent in the middle and coronal thirds in the group treated only with blood clot, whereas weak labelling was predominantly seen in these thirds in the group treated with VEGF. In addition, a greater number of specimens with immunohistological negativity and well-vascularised tissue were observed in the VEGF group. No specimens from the group treated only with blood clot achieved this level of maturation at the coronal third.

The fact that this study used a vital pulp condition is noteworthy. Then, further studies using necrotic conditions should be considered. Therefore, the option of a biological endodontic treatment free of synthetic materials seems to be closer to reality. Consequently, it can state that changes in the endodontic paradigm are real.

## Conclusions

Both treatment protocols have resulted in angiogenesis of mature root canal. The VEGF contributed positively to this process by faster revascularisation.
